# Ocular Manifestations in Atypical Hemolytic Uremic Syndrome Treated With Ravulizumab: A Case Report and Review of the Literature

**DOI:** 10.7759/cureus.84271

**Published:** 2025-05-17

**Authors:** Konstantinos Flindris, Eleni Papafotiou, Elena Mylona, Chrysa Chatzipetrou, Athanasios Kaliardas, Ioannis Koumpoulis, Ioannis Melissourgos

**Affiliations:** 1 Ophthalmology, General Hospital of Ioannina "G. Hatzikosta", Ioannina, GRC; 2 Medical School, Aristotle University of Thessaloniki, Thessaloniki, GRC

**Keywords:** ahus, atypical hemolytic uremic syndrome, hemolytic uremic syndrome, hus, hypertensive retinopathy, ocular manifestations

## Abstract

This study aims to report the ocular manifestations in a patient with atypical hemolytic uremic syndrome (aHUS), treated with ravulizumab, highlighting the reversibility of hypertensive retinal changes.

We present a retrospective case report of a 27-year-old man diagnosed with aHUS, who underwent comprehensive systemic evaluation after a respiratory infection and a hypertensive crisis. Despite the absence of ophthalmologic complaints, ocular examination revealed grade IV hypertensive retinopathy accompanied by exudative retinal detachment.

The patient received a combination of hemodialysis, intensive antihypertensive therapy, and ravulizumab. Remarkably, within one week of treatment initiation, a significant decrease in papilledema and a complete resolution of the exudative retinal detachment were observed.

This case highlights the potential for rapid reversal of severe ocular manifestations associated with systemic microangiopathic disorders, such as aHUS. The striking improvement in retinal pathology suggests that the early and aggressive management, including complement inhibition with ravulizumab, may play a crucial role in restoring ocular integrity. Furthermore, the findings advocate for routine ophthalmologic screening in aHUS patients, even in the absence of visual symptoms, to enable prompt identification and intervention in subclinical retinal involvement. Interdisciplinary collaboration between nephrology and ophthalmology is essential to optimize both systemic and ocular outcomes in such complex cases.

## Introduction

Hemolytic uremic syndrome (HUS) is a rare thrombotic microangiopathy characterized by the triad of hemolytic anemia, thrombocytopenia, and acute renal failure. HUS is classified into typical HUS (tHUS) and atypical HUS (aHUS) based on distinct etiology and pathophysiological mechanisms [1]. tHUS is predominantly caused by an infection with Shiga-like toxin-producing *Escherichia coli* (STEC) (85%-95%), particularly *E. coli* O157:H7, and is often presented in children as an enterocolitis epidemic with bloody diarrhea. The overall annual incidence of tHUS is 1-2 cases per 100,000 in the United States and Europe [2].

In contrast, aHUS is an uncommon disorder caused by genetic mutations, including those in CFH, CHI, and MCP genes, leading to the dysregulation of the alternative complement pathway. This results in hyper-activation of the C3 convertases and loss of complement regulatory mechanisms. While aHUS may be triggered by infections, autoimmune syndromes, drug response, or pregnancy, it is not associated with STEC infection. The global annual incidence of aHUS is 1-2 cases per 10,000,000 [3]. Eculizumab, a monoclonal antibody targeting complement protein C5, has been the standard treatment of aHUS since 2011 [4]. Recently, ravulizumab, a long-acting humanized monoclonal antibody C5 inhibitor, has been approved for aHUS treatment with promising results [5].

The clinical manifestations of tHUS and aHUS are similar, potentially involving end-stage renal disease (ESRD), cardiovascular complications (stroke, cardiac failure), and central nervous system (CNS) complications (seizures, coma). Diagnosis remains challenging, as it relies primarily on clinical presentation. Ocular manifestations of aHUS include hypertensive chorioretinopathy, occlusive retinal arteritis, and Purtscher-like retinopathy, with very few cases documented in the literature to date [6].

Herein, we report the case of a 27-year-old male patient with aHUS who experienced a hypertensive crisis during a respiratory infection and underwent a routine ophthalmological examination despite being asymptomatic for ocular complications.

## Case presentation

A 27-year-old male patient was referred from the Nephrology Department for a routine ophthalmological examination following a persistent hypertensive crisis during a respiratory infection. The patient had been diagnosed with aHUS at the age of 7, confirmed through genetic testing, which identified mutations in CFH R1-R3.

At initial examination, the patient presented with severe elevated blood pressure (BP) (235/156 mmHg), severe hemolytic anemia (Hb 8.4 mg/dL), acute renal failure (urea 176 mg/dL and creatinine 8.49 mg/dL), but no thrombocytopenia (platelet 189,000/μL). A comprehensive laboratory workup was performed, including viral screening (HBV, HCV, HIV, HSV, VZV) and autoimmune markers (ANA, anti-dsDNA, p-ANCA, c-ANCA, ACE, cardiolipin, beta-2 glycoprotein, anti-GBM), all of which were negative (Table 1).

**Table 1:** Laboratory blood tests at presentation INR: international normalized ratio, AST: aspartate transaminase, ALT: alanine transaminase, γGT: γ-glutamyl transferase, ALP: alkaline phosphatase, LDH: lactate dehydrogenase, CPK: creatine phosphokinase, HDL: high-density lipoprotein, TSH: thyroid-stimulating hormone, CRP: C-reactive protein, ACE: angiotensin-converting enzyme, ANA: antinuclear antibody, p-ANCA: perinuclear antineutrophil cytoplasmic antibody, c-ANCA: cytoplasmic antineutrophil cytoplasmic antibody, ds-DNA: double-stranded DNA, anti-GBM: anti-glomerular basement membrane, RF: rheumatoid factor, HBV: hepatitis B virus, HCV: hepatitis C virus,    HIV: human immunodeficiency virus, HSV: herpes simplex virus, HZV: herpes zoster virus, RPR: rapid plasma reagin

Laboratory test	Value	Reference range
White blood cells	18.35 × 10^3^/μL	4-11 × 10^3^/μL
Neutrophils	83.4%	40-75%
Lymphocytes	10.2%	20-45%
Monocytes	5.4%	2-10%
Eosinophils	0.7%	1-6%
Basophils	0.3%	0.2-1%
Red blood cells	4.01 × 10^6^/μL	3.8-6 × 10^6^/μL
Hemoglobin	8.4 g/dL	11.8-17.8 g/dL
Hematocrit	26.6%	36-52%
Mean corpuscular volume	66.3 fL	80-96 fL
Mean corpuscular hemoglobin	20.9 pg	26-32 pg
Mean corpuscular hemoglobin concentration	31.6 pg/dL	32-36 pg/dL
Platelets	189 × 10^3^/μL	140-450 × 10^3^/μL
INR	0.97	1-1.3
Activated partial thromboplastin time	31.45 s	26-36 s
Fibrogen	373.7 mg/dL	180-350 mg/dL
D-Dimers	1.55 mg/L	0-0.5 mg/L
Troponin	109.1 ng/L	0-34.2 ng/L
CK-MB	19 IU/L	0-23 IU/L
Erythrocyte sedimentation rate	36 mm	<30 mm
Fasting blood sugar	92 mg/dL	70-115 mg/dL
HbA1c	5.6%	0-6%
Urea	176 mg/dL	0-50 mg/dL
Creatinine	8.49 mg/dL	0.8-1.4 mg/dL
Potassium	3.9 mmol/dL	3.5-5.1 mmol/dL
Sodium	140 mmol/dL	136-146 mmol/dL
Magnesium	1.95 mEq/L	1.3-2.1 mEq/L
Calcium	8.9 mg/dL	8.2-10.5 mg/dL
Total proteins	6.1 g/dL	6.2-8.4 g/dL
Albumin	3.6 g/dL	3.5-5.1 g/dL
Total bilirubin	0.95 mg/dL	0.1-1.3 mg/dL
AST	22 IU/L	5-40 IU/L
ALT	32 IU/L	5-40 IU/L
γGT	26 IU/L	8-45 IU/L
ALP	67 IU/L	35-125 IU/L
LDH	627 IU/L	120-230 IU/L
CPK	118 IU/L	0-220 IU/L
Phosphate	6.6 mg/dL	2.5-4.5 mg/dL
Uric acid	10.4 mg/dL	3.6-7.8 mg/dL
Total cholesterol	161 mg/dL	120-220 mg/dL
HDL-cholesterol	42 mg/dL	35-55 mg/dL
Triglycerides	199 mg/dL	30-160 mg/dL
Ferritin	632 ng/mL	0-300 ng/mL
TSH	1.92 μIU/mL	0.35-4.94 μIU/mL
Free T4	1.03 ng/dL	0.70-1.48 ng/dL
Free T3	107.6 ng/dL	64-152 ng/dL
CRP	0.59 mg/dL	0-0.8 mg/dL
C3	84 mg/dL	79-152 mg/dL
C4	21.7 mg/dL	16-38 mg/dL
IgG	1040 mg/dL	751-1560 mg/dL
IgM	88.4 mg/dL	46-304 mg/dL
IgA	298 mg/dL	82-453 mg/dL
ACE	45 mg/dL	0-53 mg/dL
ANA	Negative	
p-ANCA	Negative	
c-ANCA	Negative	
Anti-ds-DNA	Negative	
Anti-cardiolipin, anti-beta-2 glycoprotein, anti-GBM	Negative	
RF	Negative	
Anti-HBV/HCV/HIV/HSV/VZV IgM	Negative	
RPR	Negative	

**Figure 1:**
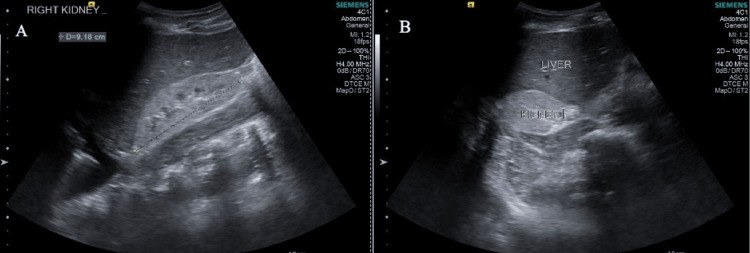
Renal ultrasound in different views: (A) transverse section and (B) vertical section

**Figure 2:**
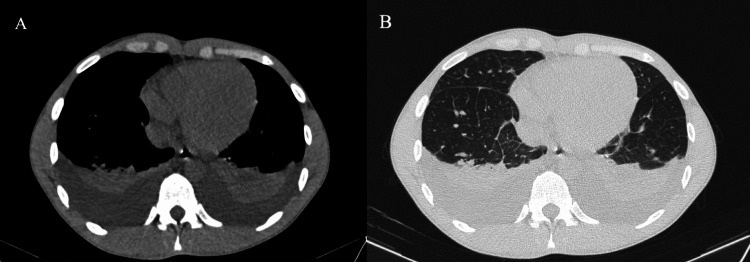
CT of the thorax, presenting bilateral pleural fusion, in different views: (A) transverse section on a lung window and (B) transverse section on a soft tissue window

Diagnostic imaging revealed multiple abnormalities. Renal ultrasonography showed findings consistent with chronic kidney failure, including reduced kidney size and increased echogenicity (Figure 1). CT of the thorax reported bilateral pleural effusion (Figure 2), while brain MRI findings were normal. Treatment was initiated with hemodialysis, antihypertensive treatment (amlodipine 10 mg/day, irbesartan 300 mg/day, furosemide 40 mg/day) and ravulizumab (2700 mg as a loading dose intravenously (IV), followed two weeks later by a 3300 mg initial maintenance dose IV, and then the same maintenance dose IV every eight weeks thereafter). The patient was fully vaccinated and had no prior history of eculizumab administration.

**Figure 3:**
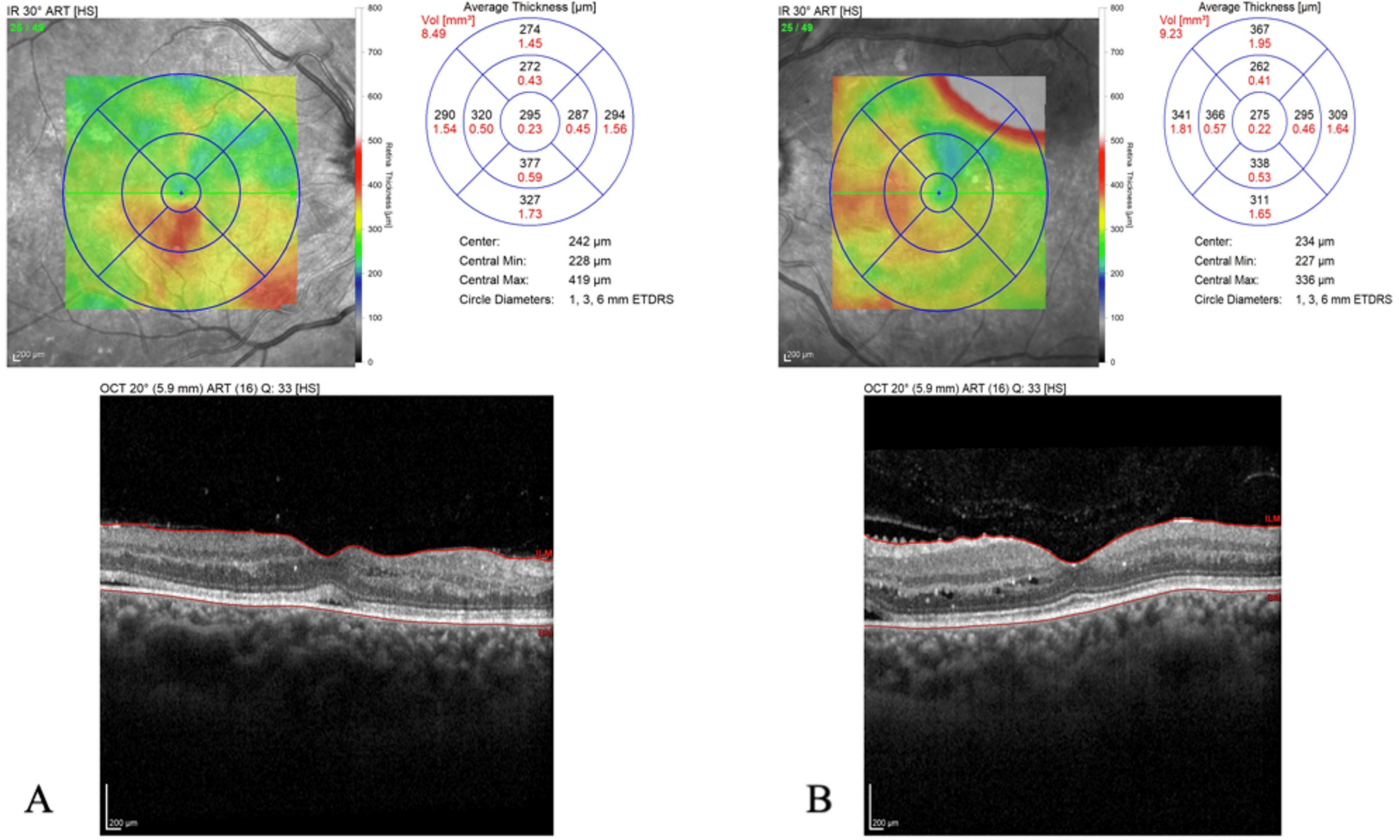
OCT in the (A) right eye and (B) left eye at presentation A horizontal scan in the macula revealed several hard exudates in both eyes and small intraretinal cysts in the left eye. OCT: optical coherence tomography.

**Figure 4:**
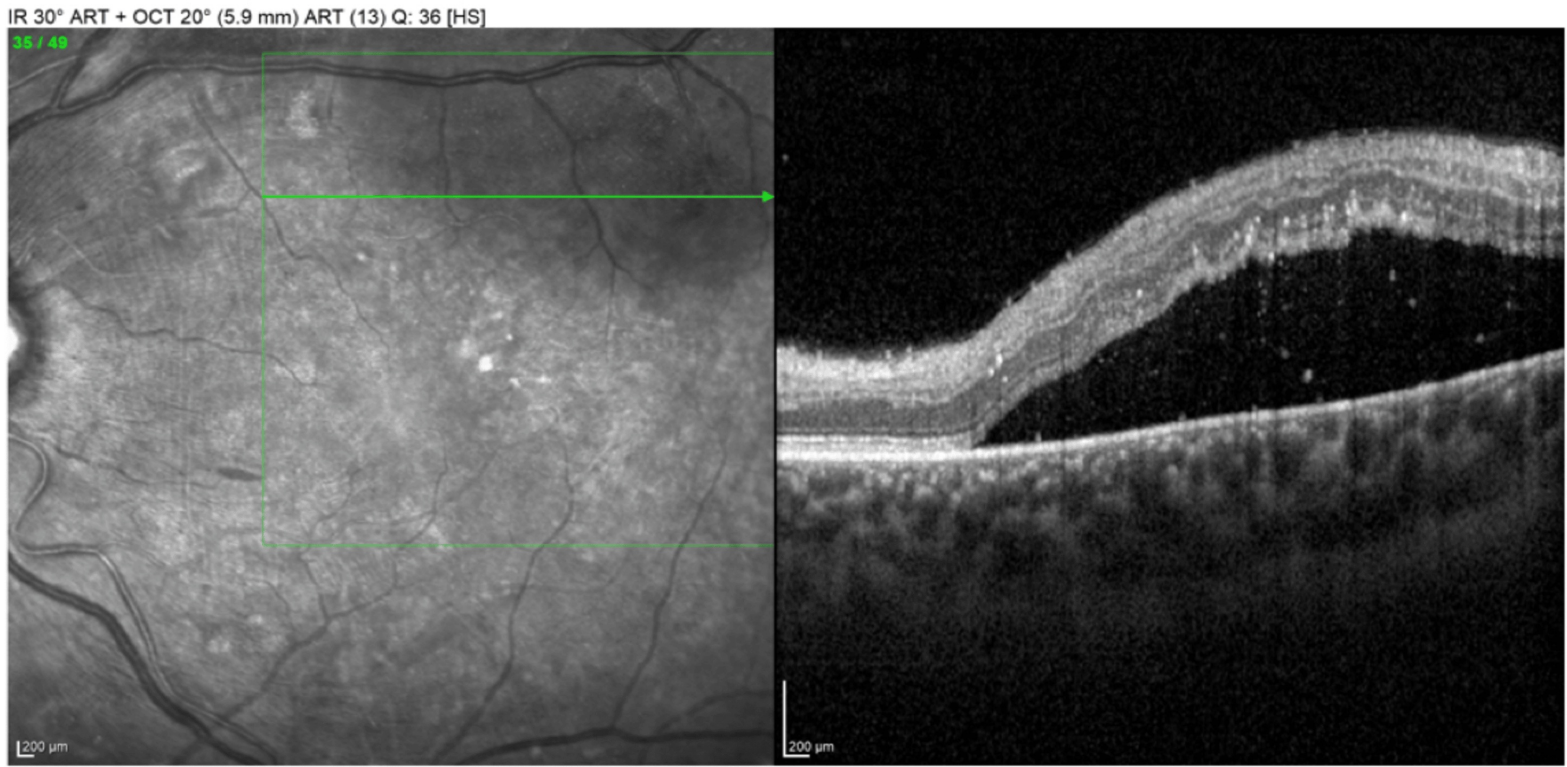
OCT in the left eye at presentation A horizontal scan presented exudative retinal detachment superior to the fovea.

The initial ophthalmological examination revealed the following findings: best corrected visual acuity (BCVA) 20/20 in both eyes, with intraocular pressure of 14 mmHg bilaterally. No relative afferent pupillary defect was observed, and both anterior segment and vitreous examinations were unremarkable. Ocular movements were normal with no diplopia. Dilated fundus examination presented bilateral hypertensive retinopathy characterized by dispersed retinal arterial narrowing, cotton wool spots, flame-shaped retinal hemorrhages, Elschnig’s spots, and papilledema. Furthermore, exudative retinal detachment was reported in the left eye. Optical coherence tomography (OCT) demonstrated several hard exudates in both eyes and small intraretinal cysts in the left eye (Figure 3), and the examination confirmed exudative retinal detachment in the left eye (Figure 4).

**Figure 5:**
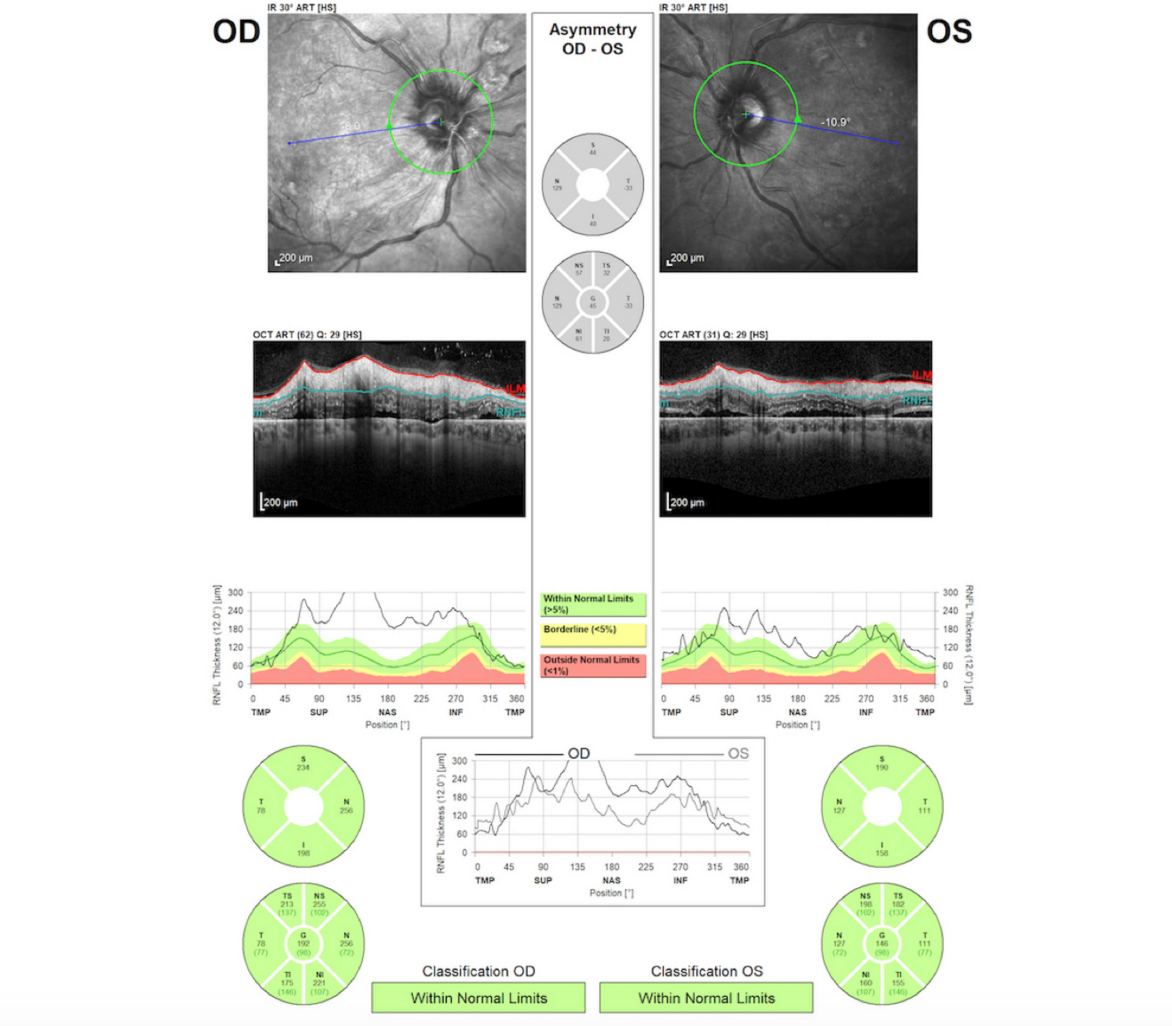
OCT of the RNFL at presentation, showing bilateral papilledema OCT: optical coherence tomography, RNFL: retinal nerve fiber layer.

**Figure 6:**
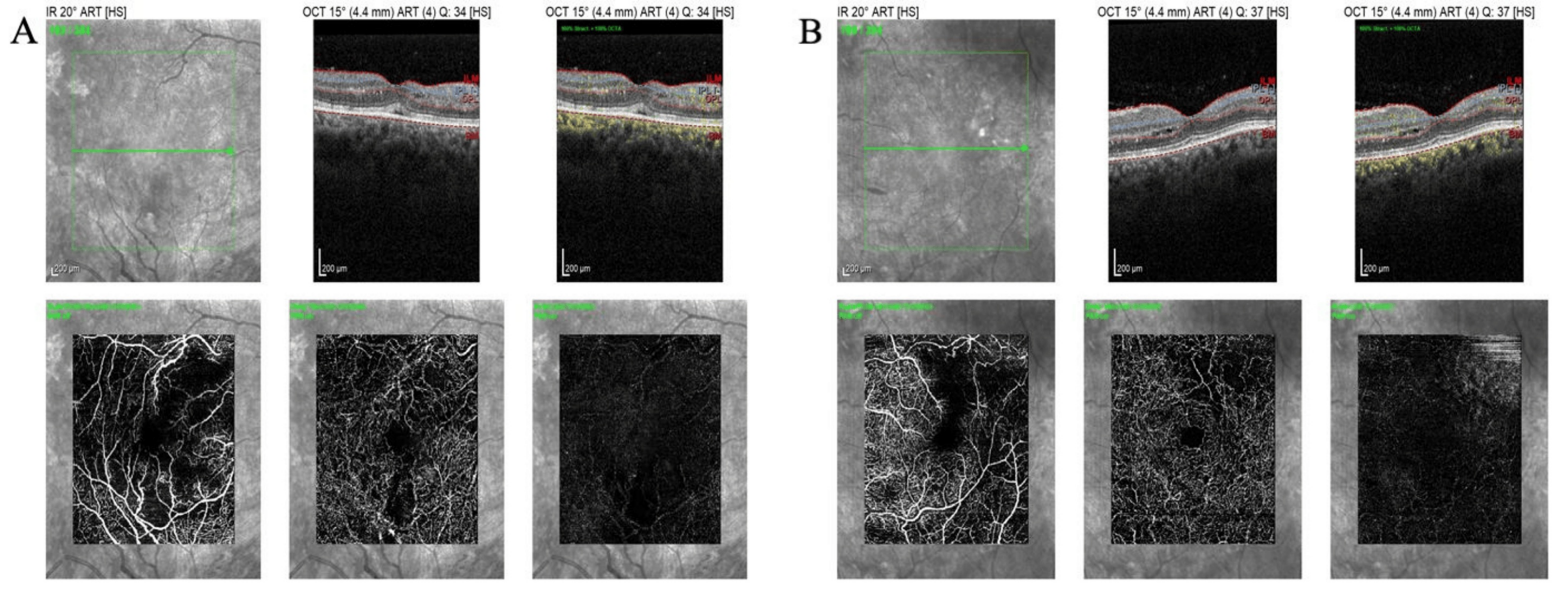
OCT-A at presentation, revealing areas of retinal ischemia in the posterior pole in the (A) right eye and (B) left eye OCT-A: optical coherence tomography angiography.

**Figure 7:**
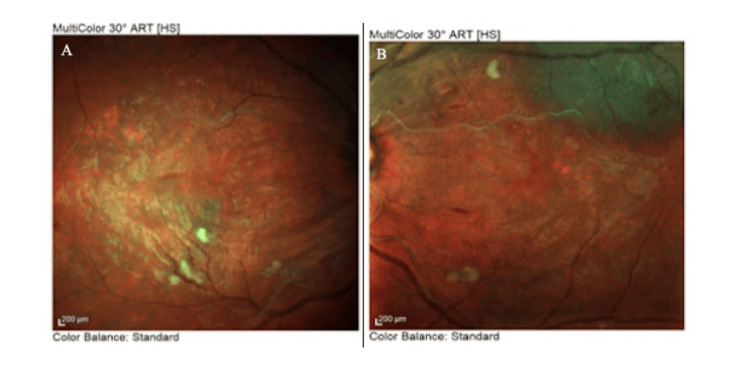
Multi-color photography of the fundus in the (A) right eye and (B) left eye at presentation

OCT of the retinal nerve fiber layer (RNFL) also confirmed bilateral papilledema, which was more severe in the right eye (Figure 5). OCT-angiography (OCT-A) presented areas of retinal ischemia in the posterior pole (Figure 6). Multi-color photography of the fundus displayed the lesions of hypertensive retinopathy in the posterior pole in both eyes (Figure 7).

**Figure 8:**
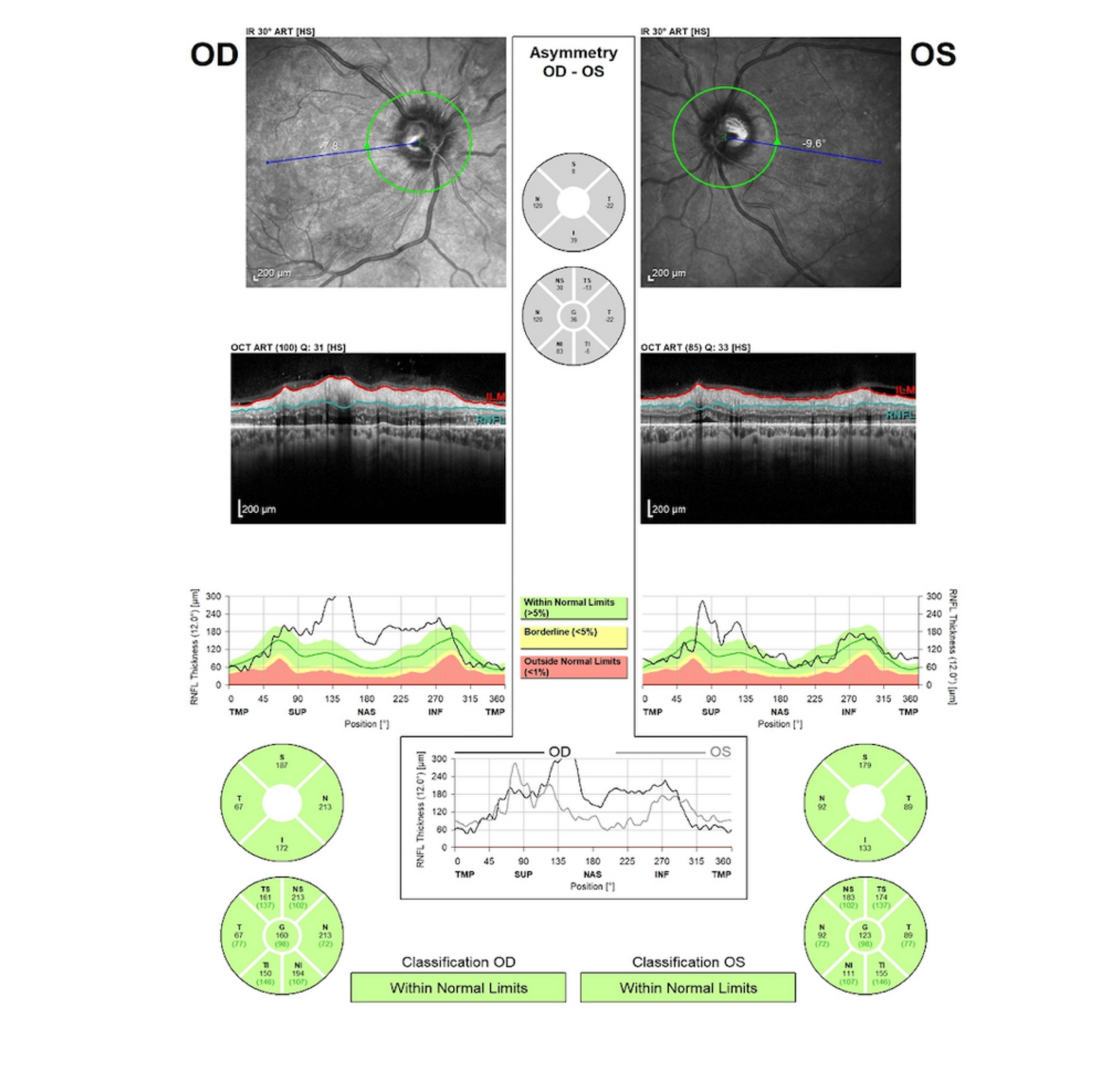
OCT of the RNFL at one-week follow-up OCT: optical coherence tomography, RNFL: retinal nerve fiber layer.

**Figure 9:**
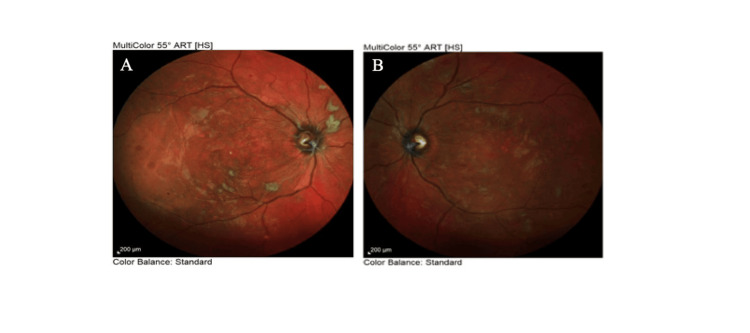
Multi-color photography of the fundus in the (A) right eye and (B) left eye at one-week follow-up

**Figure 10:**
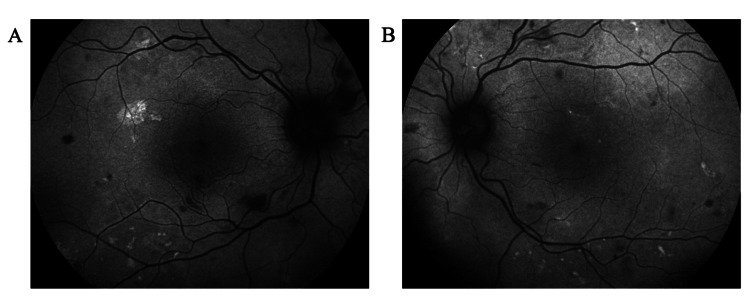
Autofluorescence imaging in the (A) right eye and (B) left eye at one-week follow-up

One week after the initial ophthalmological examination and ravulizumab administration, the patient’s BP decreased to 164/92 mmHg, while severe hemolytic anemia (Hb 8.8 mg/dL) and acute renal failure (urea 197 mg/dL and creatinine 8.67 mg/dL) persisted. BCVA remained 20/20 in both eyes, with bilateral intraocular pressure of 12 mmHg. Examination of the anterior segment, vitreous, and ocular movements was unremarkable. Dilated fundus examination showed improvement in bilateral retinal findings, including regression of arterial narrowing, cotton wool spots, flame-shaped hemorrhages, and Elschnig's spots. Additionally, the papilledema demonstrated remarkable improvement bilaterally (Figure 8), and the exudative retinal detachment resolved completely in the left eye (Figure 9). Autofluorescence imaging revealed hypoautofluorescence of multiple areas, corresponding to subretinal fluid, and hyperautofluorescence of different areas, due to intraretinal hemorrhages (Figure 10).

One month after the initial ophthalmological examination, BCVA was 20/20 in both eyes, without signs of hypertensive retinopathy in the dilated fundus examination, and the patient was monitored at regular intervals.

## Discussion

aHUS is a rare and life-threatening disorder characterized by a significant mortality rate of up to 25% during initial presentation, with outcomes varying based on the underlying genetic mutations involved in complement dysregulation. While renal manifestations are predominant in aHUS, extrarenal involvement is observed in approximately 20% of cases. Ocular complications represent a particularly rare subset of documented manifestations in the literature [7]. González et al. [8], Greenwood [9], and Sampedro Lopez et al. [10] presented cases of occlusive retinopathy, and Polito et al. [11] presented a case of hypertensive choroidopathy in aHUS.

Hypertensive retinopathy manifests due to both acute and chronic elevations in BP. Sustained hypertension compromises the integrity of the blood-retinal barrier, resulting in plasma leakage and the formation of retinal exudates. This process leads to the accumulation of fluid and blood within the retinal layers [12]. During the ischemic phase, patients may exhibit cotton-wool spots caused by infarctions of the NFL, endothelial alterations with microaneurysm formation, and retinal hemorrhages. Advanced or chronic cases can progress to optic nerve edema [13].

The condition is classified into four grades: Grade I, no clinically apparent signs; Grade II (mild), characterized by arteriolar narrowing, arteriovenous nicking, and the presence of silver or copper wiring; Grade III (moderate), includes hemorrhages, microaneurysms, cotton-wool spots, and hard exudates; and Grade IV (malignant), involves features of moderate retinopathy accompanied by optic disc swelling in the context of severely elevated blood pressure. Diagnosis is primarily achieved through a dilated fundoscopic examination. Treatment focuses on lowering BP to mitigate disease progression [14].

aHUS is characterized by dysregulated systemic complement activation, primarily driven by underlying genetic mutations. Prior to the introduction of eculizumab as a treatment option, the prognosis for aHUS was grim, with 79% of patients succumbing to death or progressing to ESRD within three years of diagnosis [15]. Both eculizumab and ravulizumab have significantly improved the outlook for aHUS by providing effective and targeted inhibition of the complement system [16]. Ravulizumab is generally preferred due to its extended dosing interval (every eight weeks compared to eculizumab’s biweekly schedule), though eculizumab continues to be an essential treatment option [17,18].

In this case, a 27-year-old male patient with aHUS presented with grade IV hypertensive retinopathy and exudative retinal detachment, due to persistent malignant arterial hypertension. Management included antihypertensive therapy, hemodialysis, and administration of ravulizumab. One week following the initial assessment and ravulizumab administration, there was complete resolution of papilledema in the left eye and regression of the exudative retinal detachment. However, renal function showed minimal recovery after one month of the initial presentation.

This case illustrates how severe retinal complications related to aHUS can be rapidly reversed with timely, targeted treatment. The swift resolution of retinal lesions, including marked improvement of papilledema and complete resolution of exudative retinal detachment, following the initiation of comprehensive therapy, underscores the critical role of early intervention. The use of ravulizumab, in combination with hemodialysis and aggressive antihypertensive management, appears to contribute significantly to restoring ocular integrity. Moreover, these findings advocate for proactive ophthalmologic screening in patients with aHUS, even when they do not report visual disturbances. Early detection of subclinical ocular changes can facilitate prompt and effective treatment, ultimately safeguarding vision and enhancing overall patient outcomes. This case reinforces the necessity for close interdisciplinary collaboration between nephrology and ophthalmology, ensuring that both systemic and ocular aspects of aHUS are addressed concurrently for optimal patient care.

## Conclusions

In conclusion, we presented an exceptionally rare case of a 27-year-old male patient with aHUS and hypertensive retinopathy and exudative retinal detachment during a hypertensive crisis without ocular symptoms. The management of aHUS has been revolutionized by complement inhibitors such as eculizumab and ravulizumab, which effectively target the dysregulated complement activation underlying the disease. Early recognition and intervention, particularly in cases complicated by hypertensive choroid-retinopathy, are paramount to preventing irreversible damage. Prompt and aggressive BP control and appropriate use of complement inhibitors can significantly improve clinical outcomes, demonstrating the importance of a multidisciplinary approach in diagnosing and managing aHUS and its complications.
